# Spanish Influenza in Japanese Armed Forces, 1918–1920

**DOI:** 10.3201/eid1304.060615

**Published:** 2007-04

**Authors:** Akihiko Kawana, Go Naka, Yuji Fujikura, Yasuyuki Kato, Yasutaka Mizuno, Tatsuya Kondo, Koichiro Kudo

**Affiliations:** * International Medical Center of Japan, Tokyo, Japan

**Keywords:** Spanish flu, pandemic, influenza, Japan, army, medical charts, Russia, research

## Abstract

With the recent outbreaks of avian influenza A (H5N1), the risk for the next influenza pandemic has increased. For effective countermeasures against the next pandemic, investigation of past pandemics is necessary. We selected cases diagnosed as influenza from medical records and hospitalization registries of Japanese army hospitals during 1918–1920, the Spanish influenza era, and investigated clinical features and circumstances of outbreaks. Admission lists showed a sudden increase in the number of inpatients with influenza in November 1918 and showed the effect of the first wave of this pandemic in Tokyo. The death rate was high (6%–8%) even though patients were otherwise healthy male adults.

## Abstract 2

Medical records of Japanese army hospitals show high death rates during the first influenza pandemic.

Because of the emergence of avian influenza A (H5N1) virus in Southeast Asia, potential evolution of a novel type of influenza in the near future is of great concern (*1**,**2*). If an outbreak of a novel form of influenza occurs, a major worldwide pandemic is predicted because humans would not have immunity against this virus. To take effective countermeasures against new pandemics, investigation of past pandemics is essential.

Four pandemics occurred in the 20th century: Spanish flu in 1918, Asian flu in 1957, Hong Kong flu in 1968, and Russian flu in 1977 (*3**,**4*). Spanish influenza was the largest pandemic, and Japan was seriously affected. Despite abundant public records related to Spanish influenza, few primary documents, such as medical records, remain in Japan. Recently, medical records from the early 20th century were found in the depository of the International Medical Center of Japan (IMCJ) Hospital, Tokyo. We used these records to investigate the clinical characteristics of Spanish influenza. To help prepare countermeasures, we investigated the outbreak situation, clinical findings, and outcomes of Spanish influenza in the Japanese military between 1918 and 1920.

## Patients and Methods

The documents were stored at the medical history depository of IMCJ Hospital, at the medical records and hospitalization registries of Tokyo First Army Hospital (a predecessor of IMCJ Hospital), and at the Fifth Japanese Army Garrison Hospital, Krasnoyarsk, Russia. Medical records in which influenza was diagnosed between January 1918 and December 1920 were selected. Because the influenza virus had not yet been discovered at that time, no serologic or virologic diagnostic methods for influenza infection were available, and no examinations, such as chest radiographs, were performed. Thus, the diagnosis flu (*kanbo* in Japanese) was defined as clinical influenza.

Three types of documents were investigated. The first type was the hospitalization registry of Tokyo First Army Hospital, in which records of 127 patients from January 1918 through November 1918 were included. Because these records were bound, it was assumed that no records were missing. The second type was the medical records of 132 patients at the Fifth Japanese Army Garrison Hospital in Russia from March 1919 through April 1920. These records were also bound, and it was again assumed that no records were missing. The third type was the medical records of 419 patients at Tokyo First Army Hospital from January 1918 through May 1920. These records were not associated with time and were partially discontinuous, which indicated that some records (dates) were missing.

Information on the hospitals (e.g., numbers of beds and physicians) was unclear. This research was reviewed and approved by the research review boards at IMCJ Hospital. Statistical significance of between-group differences was analyzed by using the Mann-Whitney U test. A p value of <0.05 was considered statistically significant.

## Results

We first investigated hospitalization registries of Tokyo First Army Hospital from January to November 1918. These registries had the names and diagnoses of patients admitted to the hospital on a monthly basis. Numbers of patients admitted for respiratory infectious disease during this period are shown in the Figure. Cases diagnosed as pneumonia, acute bronchitis, and influenza were classified as respiratory infectious diseases. Although records of patients with tuberculosis were found, we excluded them from this study. In the 10-month period from January to October, the mean (± standard deviation) monthly numbers of patients with pneumonia, acute bronchitis, and influenza were 10.9 ± 6.5, 10.0 ± 3.6, and 1.8 ± 4.1, respectively (22.7 ± 9.6 for all 3 illnesses). Death rates from pneumonia, bronchitis, and influenza during this period were 3.4% (4/116), 0% (0/109), and 0% (0/18), respectively. The number of influenza patients suddenly increased to 109 in November, and 9 of them died (8%). Because our information about these 109 patients was found only in the hospitalization registries (the patients’ medical records were not found), we could not identify their clinical symptoms. However, it can be assumed that the hospital experienced the first wave of Spanish influenza in November 1918. Because no hospitalization registry before this period was found, comparison with outbreaks in average years was not possible.

**Figure:**
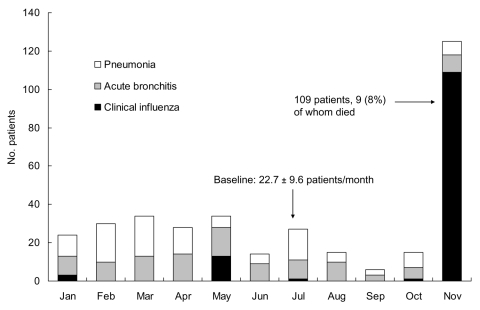
Number of patients hospitalized for respiratory infection, Tokyo First Army Hospital, 1918.

We then investigated the medical records of the Fifth Japanese Army Garrison Hospital, which may have been a Japanese military field hospital in Krasnoyarsk, Russia. These records covered 2 years (1919 and 1920), but most cases of clinical influenza were concentrated in May–November 1919. All 132 cases were in infantrymen 19–49 years of age (mean ± standard deviation 22.7 ± 4.1 years); 47% had been farmers before entering the military, and 72% had no recorded medical histories. The initial symptom was sudden fever in 116 patients (94%), headache in 101 (81%), chills in 92 (74%), cough in 86 (69%), general malaise in 60 (48%), appetite loss in 56 (45%), pharyngalgia in 45 (36%), arthralgia/myalgia in 33 (27%), and vomiting/diarrhea in 27 (22%). On admission, reddish pharynx was noted in 105 (85%), coated tongue in 95 (77%), thoracic rale in 71 (57%), facial flush in 24 (19%), and conjunctival congestion in 16 (13%). The period from onset to admission was 4.7 ± 3.9 days (range 0–24 days), the duration of hospitalization was 14.8 ± 12.0 days (range 3–79 days), and the death rate was 6.0% (8/132). A comparison of 124 patients who survived and 8 who died is shown in the Table. In the fatal cases, body temperature and pulse rate were higher at the time of hospital admission, and thoracic rale and an “agonized facial expression” (a painful expression as reported by Japanese doctors) were observed in many patients. The death rate was higher in patients who had digestive symptoms, but the difference was not statistically significant. In fatal cases, the patients died an average of 13.9 days after symptom onset and 7.1 days after admission.

Fever patterns in 132 patients were classified as typical (fever resolved in <10 days after onset), prolonged (fever persisted for ≥10 days), biphasic (fever resolved and then recurred), and other (unclassifiable). A total of 77 (58%), 38 (29%), 7 (5%), and 10 (8%) patients, respectively, were classified as these types.

## Discussion

Once a pandemic occurs, it may cause large-scale effects worldwide; various countries and organizations, including the World Health Organization, must prepare for such a situation (*5**,**6*). To take countermeasures against pandemics, past pandemics should be further investigated. Researchers are particularly interested in the biggest pandemic of the 20th century, Spanish influenza in 1918. Influenza A (H1N1) virus was the causative agent of Spanish influenza. However, this pandemic occurred almost 90 years ago, which makes investigation difficult.

Virus genes have been isolated from lung samples of patients who died of Spanish influenza. The nucleotide sequences of these genes have been determined, and the viral characteristics have been reported (*7**,**8*). However, the only method that can clarify the pathophysiology of Spanish influenza is investigation of the medical records at the time of the pandemic. Thus, the discovery of medical records and hospitalization registries of the Spanish influenza pandemic is useful. Although we studied only patients in Japan, studying patients in the Japanese Army Garrison hospital in Russia may provide a clue to the conditions of the influenza outbreak in that region.

Many theories exist as to the source of Spanish influenza, but the first reported case in the United States likely occurred in March 1918 (*3*). After that, it spread worldwide through 1920. Of the 1.8–2.0 billion persons in the world at that time, 600 million were affected and 20–40 million died from this disease (*9*). In Japan, 23 million persons were affected, and 390,000 died. The first wave occurred in Japan in August 1918, and many cases were reported in Tokyo in mid-October (*10**,**11*). Hospitalization registries of the Tokyo First Army Hospital showed a sudden increase in admissions for influenza in November 1918; this outbreak may have been the beginning of the Spanish influenza pandemic in Japan.

Because the patients were all soldiers in the Japanese army, they were essentially healthy men. Medical records indicated that the soldiers did not go home for long periods and lived together in barracks without external contact. Once a virus infection occurred, it may have caused an outbreak among the soldiers within a short time. Initial symptoms were fever, headache, chills, and cough, and their frequency was not different from those of patients with seasonal or ordinary influenza. When cases in patients who survived were compared with those in patients who died (Table), high fever, tachycardia, thoracic rale, and an “agonized facial expression” were associated with poor outcomes. All treatments were antisymptomatic supportive therapy, and none of the drugs used was typical of modern medical care. Thus, most cases may not have been affected by treatments. The duration of hospitalization was shorter for patients who died, perhaps due to rapid exacerbation of symptoms and discharge within a short time.

**Table:** Characteristics of patients who survived influenza with those who died of influenza, Fifth Japanese Army Garrison Hospital, 1919–1920*

Characteristic	Patients who survived (n = 124)	Patients who died (n = 8)	p value
Age, y	22.7	22.8	>0.9
Medical history	31/116	2/8	>0.9
Body temperature at time of hospitalization, °C	38.7	39.9	0.0005
Heart rate, beats/min	89	106	0.004
Rales	63/116	8/8	0.01
Reddening of throat	98/116	7/8	>0.9
Digestive symptoms	23/116	4/8	0.07
“Agonized facial expression”†	5/116	3/8	0.009
No. days from onset of illness to hospitalization	4.5	6.8	0.14
No. days hospitalized	14.8	7.1	0.04

*Mann-Whitney U test was used to test differences between the 2 groups. Fisher exact test was used to test differences in the ratio of the cross-calculation table. One case (at day 79) was excluded. This patient was admitted to the hospital with flulike symptoms but died of a bacterial infection. †A painful expression as reported by Japanese doctors.

Frequencies of hemoptysis and bloody sputum were reportedly high in patients with Spanish influenza (*12*), but high frequency of these signs was not observed in these patients, perhaps because of the limitation of our investigation to symptoms at the time of hospital admission. Bloody sputum was noted during hospitalization for some patients, but these episodes were excluded from the analysis because many descriptions of it during hospitalization were difficult to read. Because chest radiographs were not taken, we cannot discuss complications of pneumonia and its characteristics. However, chest auscultation indicated rales in 57% of the patients, which suggests that many patients also had pneumonia. Generally, complication by secondary bacterial pneumonia prolongs fever and leads to relapses (*13*).

To evaluate the presence of complications by bacterial pneumonia, we classified the fever patterns. Fever pattern was prolonged or biphasic in approximately one third of the patients, which suggests that many patients had secondary bacterial infection. The death rate was 6%–8%, and patients died an average of 2 weeks after onset of symptoms. The former Japanese Ministry of the Interior reported that the mean death rate from Spanish influenza in Japan was 1.21%–5.29% (*11*), but the death rate for our study patients was higher. The death rate from influenza is high for patients ≥65 years of age, but the death rate from Spanish influenza was also reported to be high for persons 20–30 years of age (*14**,**15*). The mean age of the population investigated was 22.7 years. Persons 20–30 years of age had the highest death rate and this may have been the reason for the overall higher death rate. The cause of a high death rate in young persons is unclear, but it may have been related to poor immunity because they had not previously been exposed to influenza virus. It may have also been related to poor conditions in military field hospitals at the end of World War I. Poor conditions in military field hospitals in foreign countries have also been reported (*16**,**17*).

Our study shows that when a novel influenza virus emerges, a large-scale outbreak can suddenly occur in large groups living together, such as military personnel. However, our data were incomplete because many documents were missing and radiographs were not available. Diagnoses were made solely on the basis of clinical symptoms, but this was unavoidable because no definitive virologic diagnostic techniques were available at that time.

We now have new tools against influenza, such as antiviral agents, vaccines, and rapid diagnostic methods, that were not available during the Spanish influenza pandemic. However, during the first wave of an outbreak of a new form of influenza, infection would be difficult to avoid and is likely to occur. To prepare for the next pandemic, a surveillance system for early detection of an outbreak (*18*), specific vaccines and rapid diagnostic test kits, and effective treatment strategies must be developed.
